# BIGCHEM: Challenges and Opportunities for Big Data Analysis in Chemistry

**DOI:** 10.1002/minf.201600073

**Published:** 2016-07-28

**Authors:** Igor V. Tetko, Ola Engkvist, Uwe Koch, Jean‐Louis Reymond, Hongming Chen

**Affiliations:** ^1^ Helmholtz Zentrum München – German Research Center for Environmental Health (GmbH)Institute of Structural BiologyIngolstädter Landstraße 1, b. 60wD-85764NeuherbergGermany; ^2^ BIGCHEM GmbHIngolstädter Landstraße 1, b. 60wD-85764NeuherbergGermany; ^3^ Discovery SciencesAstraZeneca R&D GothenburgPepparedsleden 1MölndalSE-43183Sweden; ^4^ Lead Discovery Center GmbHOtto-Hahn Strasse 15Dortmund44227Germany; ^5^ Department of Chemistry and BiochemistryUniversity of BernFreiestrasse 33012BernSwitzerland

## Abstract

The increasing volume of biomedical data in chemistry and life sciences requires the development of new methods and approaches for their handling. Here, we briefly discuss some challenges and opportunities of this fast growing area of research with a focus on those to be addressed within the BIGCHEM project. The article starts with a brief description of some available resources for “Big Data” in chemistry and a discussion of the importance of data quality. We then discuss challenges with visualization of millions of compounds by combining chemical and biological data, the expectations from mining the “Big Data” using advanced machine‐learning methods, and their applications in polypharmacology prediction and target de‐convolution in phenotypic screening. We show that the efficient exploration of billions of molecules requires the development of smart strategies. We also address the issue of secure information sharing without disclosing chemical structures, which is critical to enable bi‐party or multi‐party data sharing. Data sharing is important in the context of the recent trend of “open innovation” in pharmaceutical industry, which has led to not only more information sharing among academics and pharma industries but also the so‐called “precompetitive” collaboration between pharma companies. At the end we highlight the importance of education in “Big Data” for further progress of this area.

## Introduction

The Wikipedia definition “Big Data”^1^ is a term for data sets that are so large or complex that traditional data processing applications are inadequate also highlights that not only the size but also the data complexity is very important. In pharmaceutical research area, “Big Data” is emerging from the rapidly growing genomics data thanks to the rapid development of gene sequencing technology. Likewise people start to ask the question if there is Big Data in chemistry?

Over the past decade there actually has been a remarkable increase in the amount of available compound activity and biomedical data.^2^, ^3^, ^4^ The definition of “Big Data” in chemistry is generally not clear. Frequently, the “Big Data” in chemistry refers to considerably larger databases than commonly used ones (in orders of magnitude),^5^ which become recently available thanks to the emerging of new experimental techniques such as high throughput screening, parallel synthesis etc.,^3^,^6^ or as access to new chemical information as result of automatic data mining (e.g., patents, literature or in house data collections).^7^,^8^ How to efficiently mining the large scale of data in chemistry becomes an important problem for the future development of the chemical industry including pharmaceutical, agrochemical, biotechnological, fragrances, and general chemical companies.

Big Data collected from literature usually are quite noisy and the reasons are multiple. First of all, this could be due to the biological assay itself, for example the original experiment errors, assay artifacts in screening etc. Secondly, there lack of a standard way for annotating biological endpoints, mode of action and target identifier. Thirdly, errors exist when extracting data values, units and/or chemical name recognition for automatic literature mining. Some actions have been done to address these problems, e.g., improving data quality by applying promiscuity filters to clean up the screening data, developing bioassay ontology (BAO) tools to better organize and/or standardize the collected data etc.^9^, ^10^, ^11^

In order to support data analysis, collection, sharing, and dissemination several large projects, such as ELIXIR (https://www.elixir‐europe.org), eTox (http://www.etoxproject.eu), BIGCHEM (http://bigchem.eu) and others, were initiated under the sponsorship of European Commission. Many of these activities are within the core area of chemoinformatics. The BIGCHEM project was recently sponsored by EU Horizon2020 program. The consortium includes academia, big pharma companies, large academic societies (Helmholtz, Fraunhofer) and Small and Medium Enterprises (SMEs). This project mainly aims to develop computational methods specifically for Big Data analysis. Below, we briefly review challenges and opportunities in the Big Data analytics area particularly focusing on several aspects, which are going to be addressed in BIGCHEM project.

## Data Repositories

Publicly available databases such as PubChem,^3^ BindingDB,^6^ and ChEMBL^4^ (Table 1) represent examples of large public domain repositories of compound activity data. ChEMBL and BindingDB contain manually extracted data from tens of thousands of articles. PubChem was originally started as a central repository of High Throughput (HTS) screening experiments for the National Institute of Health's (USA) Molecular Libraries Program but also incorporates data from other repositories (e.g., ChEMBL and BindingDB). Commercial databases, such as SciFinder, GOSTAR and Reaxys (Table 1) have accumulated a large amount of data collected from publications and patent data. Similarly to public and commercially available repositories, industry has produced large private collections. For example, more than 150M data points are available as part of AstraZeneca International Bioscience Information System (AZ IBIS) just for experiments performed before 2008.^8^ The data quality in databases can significantly vary depending on data source, data acquisition procedures and curation efforts. Accumulated chemical patents represent another rich resource for chemical information. Large‐scale text mining has been done on patent corpus to extract useful information. IBM has contributed chemical structures from pre‐2000 patents in PubChem.^12^ SureChEMBL database^13^ was launched in 2014 providing the wealth of knowledge hidden in patent documents and currently contains 17 million compounds extracted from 14 million patent documents.

**Table 1:** Data repositories

Database	Unique compounds	Experimental		Main data types
		facts	data types		
ChEMBL v. 21^4^	1,592,191	13,968,617	1,212,831	PubChem HTS assays and data mined from literature
BindingDB^6^	529,618	1,207,821	6,265	Experimental protein‐small molecule interaction data
PubChem^3^	>60M	>157M	>1M	Bioactivity data from HTS assays
Reaxys^14^	>74M	>500M	–	Literature mined property, activity and reaction data
SciFinder (CAS)^15^	>111M	>80M	–	Experimental properties, ^13^C and ^1^H NMR spectra, reaction data
GOSTAR^16^	>3M	>24M	>5k	Target‐linked data from patents and articles
AZ IBIS^8^	–	>150M	–	AZ *in‐house* SAR data points
OCHEM^17^	>600k	>1.2M	>400	Mainly ADMET data collected from literature

Under the enormous pressure of developing new drug with more restrained R&D budget, recent years have seen large pharma companies increasingly exploring the so called “open innovation” model for drug discovery research. The collaboration between academics and pharmaceutical industry in terms of compound, data sharing has been largely increased.^18^ The examples include AstraZeneca‐Sanger Drug Combination prediction challenge to develop better algorithms for treatment of cancer.^19^ European Lead Factory^20^ is another collaboration effort of seven pharma companies, SMEs and academic partners to create a diverse library of 500k compounds by combining compounds from partners, external users and newly synthesized libraries and to screen these libraries against commercial and public targets. Both academia and industry should benefit from these kind of collaborative efforts, which can result in more chemical and biological data being available in the public domain. More interestingly, even the collaborations between pharmaceutical companies on the so‐called “precompetitive” level, which was hardly to imagine ten years ago, has become a trend. These efforts have made sharing of data within each organization become possible and lead to a further increase in the size of “Big Data”.^21^,^22^

## Frequent Hitters Analysis

Big Data sometimes also means noisy data. Data coming from HTS experiments could often be contaminated with false positive and false negative results. The errors can appear due to casual problems such as measurements errors, robotic failure, temperature differences, etc., which could be easily addressed with proper experimental protocols (e.g. by repeated measurements). Unfortunately, there are also systematic problems, such as low solubility in water, degradation of compounds or “frequent hitters” (FH). The first two problems can be solved with, e.g. proper analytical procedures, while the latter requires another type of consideration.

FHs are usually referred to as compounds that provide unspecific activity in different assays.^23^ Some of these compounds cause nonspecific binding (*e.g*., reactive compounds) or/and interfere with a particular assay technology (*e.g*., light quenching, compounds forming micelles, luciferase inhibitors, formation of complexes with tagged proteins for AlphaScreen^24^). Others are promiscuous binders that interact with different targets in a specific, dose‐dependent fashion^25^ and could constitute up to 99.8 % of hits.^26^ An analysis of results of these HTS data without filtering non‐specific binders and compounds that interfere with the assay technology could result in a model to predict FHs and not the target activity. Therefore carrying out FH analysis would help to clean screening data and eventually help to build better predictive model.

Various sources for compounds behaving as FH have been proposed such as: chelation, redox activity, membrane disruption, singlet oxygen production, compound fluorescence, cysteine oxidation and non‐selective reactivity.^26^ It was also estimated that 1–2 % of drug‐like compounds could self‐associate into colloidal aggregates that non‐specifically inhibit enzymes and other proteins at a typical screening concentration of 5 uM.^27^ Baell and Holloway looked at compounds with activity in multiple assays^11^ and found certain substructures, which appeared repeatedly in promiscuous hits, and labeled them “Pan Assay Interference Compounds (PAINS)”.

The non‐specific binding, however, can be also important and be extensively exploited by nature. A significant overlap between PAINS substructures and natural products for quinones and catechols^28^ indicate that these scaffolds were selected by evolution for their shotgun properties. Other substructures such as 2‐amino thiazoles have been shown to be FHs in the sense of promiscuous binders, but are also present in marketed drugs.^29^ An application of alerts developed by chemical providers to flag problematic compounds found that drugs are two‐three folds enriched with such alerts as compared to the screening libraries.^30^ Thus, a blind exclusion of “undesired” compounds may result in a significant risk to miss potentially interesting compounds and thus throw the baby out with the bathwater.

In order to develop FH filters it is important to find assays, which use similar technology. To be able to better analyze and compare different HTS data the Bio‐Assay Ontology (BAO) concept was developed.^10^,^31^ It has been used to both annotate HTS in PubChem and in an industrial setting.^9^ The use of BAO makes it easier to group assays according to the used technologies and to identify relevant FHs. The identified catalogue of FH substructures can be very useful to remove chemical matter in future HTS campaigns that will specifically interfere with the used assay technology. Thus, the information about the mechanism of action of FHs will be important to design and correctly interpret screening campaigns.^24^,^32^

It should be noted that even the best BAO and best methods of standardization of experiments will never fully address the problem of heterogeneity and complexity of biological and chemical data. By no means experiments performed in mouse and in rats can be combined into one single “activity” column associated to a single, standardized structure for all possible experiments. Such merge can be performed only depending on the conditions of experiments, endpoint and, importantly, properties of compounds. For example, even for simple lipophilicity property logP shake‐flask values can be merged with logD values obtained from HPLC experiments only for compounds non ionized under the pH of the experiment but not for all possible structures.

## Data Visualization and Exploration of Chemical Space

The visualization and compact representation of millions of compounds (such as >110M compounds in SciFinder, see Table 1), which is usually the first step of data analysis, represents significant challenge in Big Data analysis. It is usually done by projecting large compound collections into a low dimensional space, amenable to visual inspection and intuitive analysis by the human brain. It could help to detect chemical entities with novel chemical scaffolds and physicochemical properties (*e.g*., for compound library design), to compare different libraries or to identify regions of chemical space that possess certain pharmacological profile.^33^ Exemplary approaches such as principle component analysis (PCA),^34^ Generative Topographic Mapping (GTM),^35^ Kohonen networks,^36^ Diffusion Maps,^37^ and interactive maps obtained by projection of high‐dimensional descriptor spaces,^38^,^39^ are promising techniques in this context. Such visualization methods can be also used to interpret structure‐activity relationships.^40^ For example, in the “Stargate” version of GTM, latent space links two different initial spaces – one defined by molecular descriptor and another one by experimental activities.^41^ This allows, on one hand, to predict the whole pharmacological profile for one particular chemical structure and, on the other hand, to identify new structures corresponding to the given profile. Another example of exploring chemical space is using a so called ChemGPS approach to represent and navigate through drug‐like^42^ and pharmacokinetic^43^ chemical space based on PCA components extracted from molecular 2D descriptors. Its variant ChemGPS‐NP^44^ characterize the natural product space in particular. It has been shown that the accuracy of describing molecules in ChemGPS‐NP defined space is similar to the accuracy of structural fingerprints in retrieving bioactive molecules.^45^

Beside the space represented by known and available chemical structures, the chemical space composed by virtual compounds is much bigger. The number of potential molecular structures, which could theoretically be enumerated, is vast. For example, the database GDB‐17 contains 166.4 billion molecules that are possible combinations of up to 17 atoms of C, N, O, S and halogens following simple rules of chemical stability and synthetic feasibility.^46^,^47^ Although GDB‐17 is already very large, it would be many orders of magnitude larger if extended to 20–30 heavy atoms, which is the average size of drug‐like molecules.^48^ These data sets raise new challenges even for traditional profiling of chemical compound collections, which is used to identify chemicals with favorable properties (e.g., Lipinski's rule, non‐toxic, etc.). Even a fast algorithm, which is able to process 100,000 molecules per minute, will require >1,000 days∼3 years of calculations on one core to annotate the full GDB‐17. If the model supports efficient parallelization, it could be executed on, e.g., the supercomputer of Leibniz Supercomputing Centre with 241,000 cores. In this case the calculation time can be theoretically decreased to ten minutes. Instead of a brute force approach one can rely on, e.g. sequential triaging scheme that eliminates undesired regions, such as low solubility or low prediction accuracy due to limited applicability domain of model,^49^ by very fast algorithms first and then applies more compute expensive methods on smaller subspaces. Thus novel approaches or workflows are needed to efficiently search through this enormous chemical space.

## Structure‐Activity Relationship Modeling

Although a plethora of machine learning algorithms is available for SAR studies^50^,^51^ there is an increasing need for robust and efficient computational methods, which are able to cope with very large and heterogeneous datasets. The current methods already allow to build predictive models from hundreds of thousands of compounds and high‐dimensional descriptors with data matrices of >0.2 trillion entries.^5^ Advances in this field can be also expected from data fusion methods, which simultaneously model several related properties.^52^ The simultaneous modeling of such incompatible data by exploring inter‐correlation between different properties, e.g. tissue/air partitioning coefficients in humans and in rats, has already successfully contributed models with improved accuracy compared to those built with any single activity data.^52^ Numerous methods have been developed to predict compound polypharmacology.^53^, ^54^, ^55^ Prediction of the binding affinity of ligands to multiple proteins allows to anticipate potential selectivity issues, discover beneficial multi‐target activities as early as possible in the drug discovery process,^56^ or make target deconvolution for phenotypic screening.^57^ Most of these methods rely on building single target model individually, one future development could be to use all available chemogenomics data to pursue multi‐task learning and build one multi‐label model to predict multiple target activity simultaneously. A recent study shows that massive multitask networks obtain predictive accuracies significantly better than single‐task methods.^58^ Probabilistic matrix factorization (PMF) methods have been found particularly useful in building multi‐task model.^59^,^60^ Further injection of ligand and protein information into PMF method as side information may further improve the prediction accuracy.^61^ However in Big Data setting, this would require huge computer power and dedicated parallel programming model. Recently deep learning technology has gained large attention in public media. In 2015 the deep learning models achieved accuracy of human brain for handwritten Chinese character recognition^62^, while in 2016 a deep‐learning network won Go^63^ tournament against the human champion. Moreover, recent announcement of Google Cloud Platform^64^ has made possible the use of technologies staying behind the best implementation of machine learning algorithms by non‐experts. The deep learning neural network technology, which is able to efficiently deal with high‐dimensional and complex data, has also been applied in chemoinformatics area^51^,^65^,^66^ and is expected to further contribute to the progress of this direction of studies.

Another important question is “does more data contribute better models”? A consensus model to predict melting point (MP), which was developed with N=275k measurements, calculated RMSE=31±1 °C for Bergström data set^67^ of drugs (N=277).^5^ This result is almost 15 °C improvement compared to the results of the original study^67^ and 3 °C improvement compared to the model developed with N=47k molecules.^68^ It should be noticed that models were developed using different descriptors and machine learning methods, which could contribute to the difference in their performances. To exclude influence of these factors, we used exactly the same protocols from ref^5^ to develop a model using Bergström data only. The developed consensus model calculated RMSE=50±1 °C confirming, on one hand, that the improvement in the prediction accuracy for MP was contributed by an increase of the training set size and, on the other hand, suggesting that modern automatic text patent mining, which was used to contribute >80 % of data of the 275k set, produced data of excellent quality.

## De Novo Design

De novo design aims at generating new chemical entities with drug‐like properties and desired biological activities in a directed fashion. Comparing with normal virtual screening or HTS, which search for active compounds in physically available compound database, de novo design tries to generate hypothetical candidate compounds *in silico*. There are mainly two type of methods for making de Novo molecular design, one is the based on the similarity to known active compounds, i.e., ligand based De Novo design, and the other type is based on protein 3D structure to generate new compounds, i.e., structure based De Novo design. Here we mainly discuss ligand based methods, structure based methods can be found in elsewhere.^69^

One way for doing de Novo design is to search the large virtual compound database such as the GDB to get de novo hits. In order to search vast virtual chemical space, one would need integrated workflows combining efficient search and multiparameter optimization strategies to filter out molecules with sub‐optimal profiles as early as possible. For example physicochemical and synthetic feasibility filters can be frontloaded to trim down the number of compounds. Ruddigkeit et al.^70^ was able to search entire GDB17 database with a workflow combining MQN 2D structure fingerprint with ROCS shape matching method. Another strategy is reaction‐driven, fragment‐based de novo design. Based on known chemical reactions and commercially available build blocks, chemically diverse and synthetically feasible compounds are generated via normally multistep and multi‐parameter optimization process searching for candidate compounds which satisfy to certain property profile. These reaction‐based methods have been successfully applied to design de novo bioactive compounds.^71^, ^72^, ^73^

The third strategy to provide an intelligent search of new compounds is to generate structures, which are sufficiently new but still within the chemical spaces covered by the models. A group of these methods, which is known as “inverse QSAR”, has received a boost during recent year due to increasing computational power and new theoretical developments. A set of linear constrained Diophantine equations was used by Faulon et al.^74^ to exhaustively enumerate new compounds. Wong et al.^75^ used kernel methods to map training compounds from input space to the kernel feature space. In this space the authors generated new data points, which were used to recover the chemical structures. Actually, this approach is similar to that of aforementioned “Stargate” GTM,^41^ with an exception that the former algorithm does not use supervised learning to create maps. In another approach Funatsu et al.^76^ used Gaussian models and Bayesian inference to exhaustively fill a target region of the model space with new structures. Thus, these methods propose novel chemical structures while still staying within the chemical space of the QSAR models.

## Data Sharing and Data Security

Even large pharma companies can accumulate only limited amounts of relevant property information. As it was mentioned before, sharing data collected by different organizations offers the opportunity to develop computational models on a much broader data basis, thereby increasing model robustness, accuracy and coverage of chemical space.^77^,^78^ The development of approaches to predict ADME/T properties in a collaborative manner is becoming a part of future pharma R&D strategies. Recently, AstraZeneca and Bayer made the efforts to compare their entire compound collection in a secure manner,^22^ while AstraZeneca and Roche started a data sharing consortium on the topic of matched molecular pairs to improve metabolism, pharmacokinetics, and safety of their compounds through MedChemica.^21^ Moreover, AstraZeneca has already donated some of its ADMETox data to CheMBL.^79^ However, collaborative efforts in this field are generally not straightforward. The intellectual property aspects associated with private compound collections and associated data might be relevant for ongoing drug discovery efforts. Secure multiparty computation methods based on modern encryption theory^80^,^81^ provide ways to develop models without the need to share molecular structures or proprietary molecular representations. These methods are compute‐intense and bandwidth‐demanding but fast development of Internet and increasing computational power of computers is making them applicable to real world problems.^82^,^83^

## Training Big Data Scientists – The Chemoinformaticians

The “Big Data” challenges require professionally trained experts, “data scientists in chemistry” – the chemoinformaticians, who can cope with the complexity and diversity of problems in this field of scientific discovery. Traditional “data scientists” coming from computer science field, as well as computational chemists with little knowledge in computer science, are very unlikely to have sufficient knowledge and expertise to address both chemoinformatics questions and will need additional training. Important questions in this regard are following ones: How should one balance chemistry and computer science training? How should one ensure a high level of scientific expertise and, at the same time, a practically oriented mindset? Which new and rapidly developing methodologies should be considered? How should one prepare trainees to work at the interface between computing, chemistry, and pharmaceutical research? These questions can be answered only during close interactions of academic partners and the end‐users and tight involvement of industrial partners in targeted research trainings. In this respect the training programs, such as offered through Marie Skłodowska‐Curie Actions, provide generous funding support by means of Innovative Training Networks, which foster and promote such type of interactions.

## Conclusions

Both industry and academic partners share high expectations from “Big Data” in chemistry, which is a new emerging area of research on the borders of several disciplines. The advance in this area requires development of new computational approaches and more importantly education of scientists, who will further progress this field.

## Conflict of Interest

IVT is CEO and founder of BigChem GmbH, which licenses the OCHEM 17.
